# Discovery of the vector of visceral leishmaniasis, *Phlebotomus* (*Artemievus*) *alexandri* Sinton, 1928, in Kenya suggests complex transmission dynamics

**DOI:** 10.1016/j.crpvbd.2023.100134

**Published:** 2023-07-28

**Authors:** Steve Kiplagat, Jandouwe Villinger, Collins K. Kigen, Kevin O. Kidambasi, Jackson M. Muema, Stephie M. Mwangi, Maureen Wangari, Damaris Matoke-Muhia, Daniel K. Masiga, Joel L. Bargul

**Affiliations:** a International Centre of Insect Physiology and Ecology (icipe), Nairobi, P.O. Box 30772-00100, Kenya; b Department of Biochemistry, Jomo Kenyatta University of Agriculture and Technology, Nairobi, P.O. Box 62000-00200, Kenya; c Institute for Immunology and Infection Research, School of Biological Science, University of Edinburgh, Edinburgh, UK; d Centre for Biotechnology Research and Development, Kenya Medical Research Institute, P.O. Box 54840-00200, Nairobi, Kenya

**Keywords:** *Phlebotomus alexandri*, Sand flies, *Sergentomyia*, *Leishmania*, Cutaneous leishmaniasis, Visceral leishmaniasis, Blood meals, Kenya

## Abstract

Visceral and cutaneous leishmaniasis are endemic to specific regions due to the ecological preferences of phlebotomine sand flies and *Leishmania* spp. transmission. Sand fly entomological data in northern Kenya are scarce due to limited studies and neglect of leishmaniasis. The aim of this study was to investigate: (i) sand fly diversity and distribution; (ii) occurrence of *Leishmania* DNA within sand flies; and (iii) blood-meal sources of sand flies in Laisamis, northern Kenya. We conducted an entomological survey during February and March of 2021 in five areas of Laisamis sub-county using standard CDC light traps. A total of 1009 sand flies (394 male and 615 female) were morphologically identified, and representative samples verified by PCR amplification and sequencing of the cytochrome *c* oxidase subunit 1 (*cox*1) gene. Similarly, we identified blood-meal sources and *Leishmania* DNA in female sand flies by PCR amplicon sequencing of the vertebrate *cytochrome b* (*cyt b*) gene and internal transcribed spacer 1 (ITS1) of the 28S rRNA gene, respectively. *Sergentomyia clydei* (59.8%) was the most abundant sand fly species. Though collected mainly from one locality (Tirgamo), 14.8% of samples belonged to *Phlebotomus* (*Artemievus*) *alexandri* Sinton, 1928. We detected DNA of *Leishmania major* in 5.19% of *Ph. alexandri*, whereas *Leishmania adleri* DNA was detected in *S. clydei* (7.51%), *Sergentomyia squamipleuris* (8.00%), and *Sergentomyia africanus* (8.33%). Nine of 13 blood-fed sand flies had obtained blood from humans, of which 33.3% had *L. major* DNA. Both *Ph. alexandri* and *S. clydei* primarily fed on humans and could potentially be involved in the transmission of cutaneous leishmaniasis. The findings of this study contribute to the understanding of sand fly vector populations and their potential to transmit leishmaniasis in the area.

## Highlights

• *Leishmania major* DNA detected in *Phlebotomus alexandri* from Laisamis, Marsabit, Kenya.
• *Phlebotomus alexandri* feed on humans in Laisamis Ward.
• Areas of Laisamis Ward harbor diverse sand fly species.

## Graphical abstract

**Figure:**
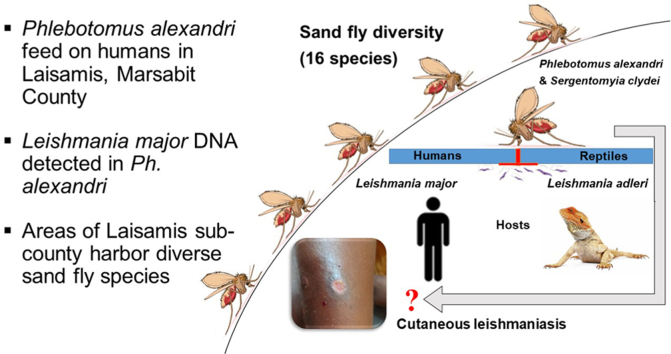

## 1 Introduction

Phlebotomine sand flies (Family Psychodidae) are small hematophagous insects (Khan et al., 2017) of public health importance because of their role in the transmission of several etiological agents of diseases, including leishmaniasis (Fernández et al., 2016). Globally, over 0.7 million leishmaniasis cases are reported annually and about 350 million people are at risk of contracting the disease across 98 countries (Jones and Welburn, 2021). In Kenya, leishmaniasis prevalence is not clear despite several outbreaks affecting children ≤ 5 years-old and young adults (KSPC, 2021).

The intracellular protozoan parasites of the genus *Leishmania* (Family Trypanosomatidae) are transmitted during blood-feeding by female sand flies of the genera *Phlebotomus* Rondani & Berte and *Lutzomyia* França, in the Old World and New World, respectively (Torres-Guerrero et al., 2017; Ngere et al., 2020). Although some members of the genus *Sergentomyia* Franca & Parrot, have been reported to mechanically spread *Leishmania* parasites during blood-meal acquisition process, they are not considered biological vectors of leishmaniasis, but rather flies of biting nuisance (Anjili et al., 2011; Owino et al., 2021). Clinically, there are four forms of the disease: visceral leishmaniasis (VL; also commonly referred to as ‘kala-azar’), which is the most fatal form of the disease; cutaneous leishmaniasis (CL); mucocutaneous leishmaniasis (MCL); and a putative post-kala-azar dermal leishmaniasis (Inceboz, 2019; WHO, 2021). Occurrence of these forms of the disease is influenced by the geographical distribution of the sand flies (Tsirigotakis et al., 2018), the ecological characteristics of the area, and human behavior (WHO, 2021) with a predilection for low socio-economic settings (Mann et al., 2021).

As in other countries of eastern Africa (Ethiopia, Uganda and South Sudan), VL is primarily caused by *Leishmania donovani* (Elnaiem, 2011), which has been known to be transmitted by only two sand fly species (*Phlebotomus orientalis* (Parrot) and *Phlebotomus martini* (Parrot) of the subgenera *Larroussius* Nitzulescu and *Synphlebotomus* Theodor, respectively) (Anjili et al., 2011). *Phlebotomus vansomerenae* (Heisch, Guggisbergr & Tesdale) and *Phlebotomus celiae* (Minter) (both belonging to subgenus *Synphlebotomus*) are suspected and probable secondary vectors of VL in limited foci of Kenya (Elnaiem, 2011). Three members of the genus *Phlebotomus*, *Phlebotomus pedifer*, *Phlebotomus guggisbergi* Kirk & Lewis (all belonging to subgenus *Larroussius*), and *Phlebotomus duboscqi* (belonging to subgenus *Phlebotomus*), have been reported as vectors of CL caused by *Leishmania aethiopica*, *Leishmania tropica* and *Leishmania major*, respectively (Owino et al., 2019). Other *Phlebotomus* sand flies incriminated in the transmission of CL in Kenya include *Phlebotomus aculeatus* Lewis, Minter & Ashford and *Phlebotomus saevus* Parrot & Martin, both of which have been reported in Gilgil in Nakuru County. *Phlebotomus saevus* was also found in other parts of Kenya, such as Samburu County in the Rift Valley and Isiolo County in eastern Kenya (Sang et al., 1993; Ngumbi et al., 2010; Anjili et al., 2011). Although *Ph. martini*, *Ph. orientalis* and *Ph. saevus* have been reported to be present in northern Kenya, along with the more common sand fly species of the genus *Sergentomyia* (Ngumbi et al., 2010; Anjili et al., 2011; Elnaiem, 2011), there is a paucity of data regarding sand fly populations and their ecological characteristics.

Various counties in northern Kenya have reported outbreaks of VL occurring almost annually in the last decade. Since 2014, Marsabit County has experienced several outbreaks of VL with a major recurrence in 2017 with 104 cases and three fatalities (Dulacha et al., 2019). Due to low index of suspicion, negligence, and poor health-seeking behavior, some cases are suspected to go unreported and could consequently contribute to risks for future outbreaks. The major contributing factors to these frequent outbreaks are not yet known, despite high morbidities and mortalities due to the disease (Kanyina, 2020).

Currently, reliable and practical approaches for controlling leishmaniasis require understanding of the sand fly vector behavior (Zivdari et al., 2018; Hassaballa et al., 2021). Blood-meal analysis of these hematophagous insects is important to identify not only the blood-meal sources, but also their preferred hosts under natural conditions (Elaagip et al., 2020). Availability of sensitive PCR-based assays and amplicon sequencing has allowed for better understanding of the cryptic nature of leishmaniasis and its transmission (Hassaballa et al., 2021). We conducted a surveillance study in Laisamis, Kenya, to understand sand fly diversity, distribution, and their potential involvement in leishmaniasis transmission in the area.

## 2 Materials and methods

### 2.1 Study area

**Fig. 1:**
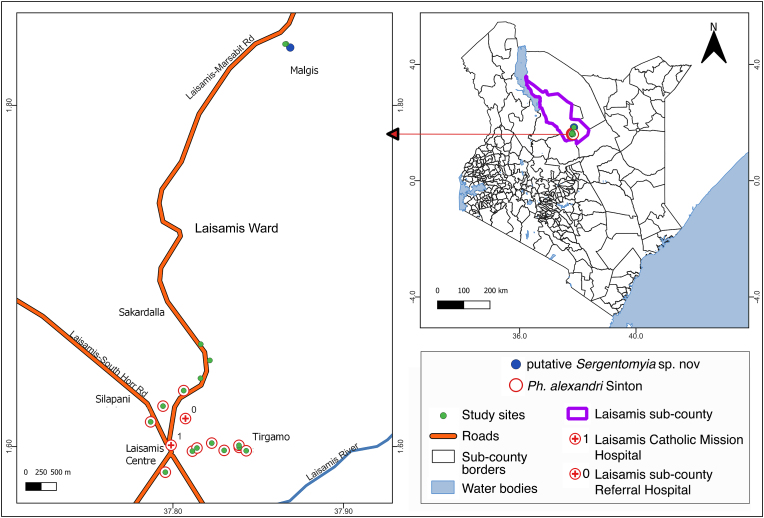
Map of Kenya showing the sampling sites in Laisamis, Marsabit County. Sand flies were sampled in Sakardalla, Silapani, Laisamis Centre, Tirgamo, and Malgis. The map was designed using QGIS v3.22.

**Fig. 2:**
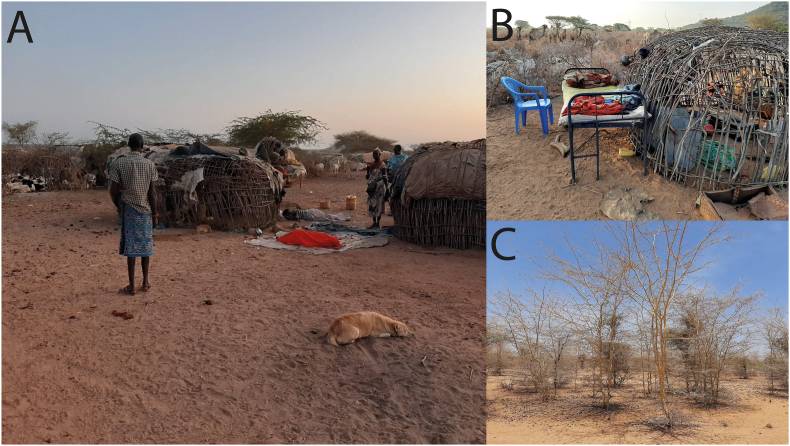
Illustrations of vegetation cover and culture of outdoor sleeping by the residents of Laisamis, Marsabit County: **A**, **B** Outdoors sleeping. **C***Acacia xanthophloea* (yellow barked) dominant vegetation in Tirgamo, Silapani, and Laisamis Centre.

This study was conducted in Laisamis Ward (1.6°N, 37.8°E, 579 m above sea level), Marsabit County, in northern Kenya (Fig. 1). Laisamis Ward occupies an area of 20,266 km^2^ with a population of about 84,056 people (KNBS, 2013). The area has an average temperature of 26.5 °C (minimum = 19 °C, maximum = 30 °C; March and July are the warmest and coldest months of the year, respectively). Although the amount of rain can range from 200 to 750 mm annually, the area receives little rainfall throughout the year (Chufe et al., 2019). Long rains frequently occur between April and June, while short rains occur between October and December. Long dry spells are experienced regularly with irregular and unpredictable climatic patterns becoming more common. Most residents belong to Rendille tribe whose main economic activity is livestock production, which include camels, sheep, goats and chicken (Bargul et al., 2021). The elevation of the selected sampling sites ranged between 472 m and 580 m above sea level, with predominant vegetative cover of *Acacia xanthophloea* and *Acacia seyal* (Fig. 2C).

Sand flies were randomly sampled in the following localities of five areas: Manyatta (village) Ntiliya in Silapani (1.61478°N, 37.78849°E); Manyatta Mbirikany, Dafardai, Naigero and Rongumo in Tirgamo (1.59769°N, 37.84256°E); Manyatta Esimatacho, Lawai and Marleni in Sakardalla (1.660026°N, 37.81629°E); and Kula Pesa, Laisamis secondary school, and Laisamis Catholic Mission Hospital in Laisamis Centre (1.59642°N, 37.81061°E). As locals had informed us of frequent *Leishmania* infections of pastoralists when grazing animals in Malgis (1.835081°N, 37.869628°E) south of Log-Logo, we included this area for sampling in this study (Fig. 1; Supplementary Table S1); it is in a tropical semi-arid area characterized by plain topography with a seasonal river crossing between hillocks of black volcanic rocks with a vegetative cover of *Commiphora holtiziana* trees.

### 2.2 Sampling of phlebotomine sand flies

Eighteen standard CDC light traps (John W. Hock Company, Gainesville, FL, USA) baited with CO_2_ dry ice were used to trap outdoor flying sand flies between sunset (18:00 h) and early morning before sunrise (5:30–7:00 h) daily for a total of 21 days during February and March of 2021. Trapping involved areas within the household perimeter, i.e. where family members sleep outdoors (most individuals were sleeping outdoor; Fig. 2A and B), and around the livestock shades. In Malgis, sand flies were trapped inside cave-like spaces in the rocky hills inhabited by rocky hyraxes. All collected sand flies from each sampling site were separately sorted out from other caught insects and preserved in 70% ethanol before being transported to a makeshift field-based laboratory where sand flies were dissected and packaged for further analysis.

### 2.3 Morphological identification of sand flies

The collected sand flies were washed in 2% colorless Teepol detergent to remove the hairs or setae and cleaned in distilled water before dissections. The heads, legs, and the last two abdominal segments of the female sand flies were dissected out using clean dissecting pins under a dissecting microscope, mounted onto labeled microscope slides using gum chloral oil (Berlese-type medium) and left to dry for 2–4 days or until dried before being morphologically identified. The remaining parts, thorax and some parts of the abdomen, were immediately preserved in liquid nitrogen and transported to the *icipe*'s Duduville campus in Nairobi where they were stored in −80 °C awaiting further analysis. For 215 female and all male sand flies, whole fly parts were mounted onto labeled slides.

The sand flies were morphologically identified based on differences in pharynx armature, gonostyle size, adeagus shape, size of the gonocoxite process, and distinctive variations in the antennal segment III (A3) length using a combination of morphological keys (Abonnenc and Minter, 1965; Lewis, 1982). Generally, *Ph. alexandri* and *Ph. saevus* were distinguished based on difference in the size of the gonocoxite process, while the females were also differentiated based on variations in A3, spermathecal segments, and pharyngeal armature. *Phlebotomus martini*, a member of subgenus *Synphlebotomus*, was distinguished based on possession of 5 major spines on the gonostyle and a short gonocoxite tuft compared to *Ph. orientalis*, a member of subgenus *Larroussius* also possessing 5 spines on the gonostyle but lacking gonocoxite tuft on the coxite.

### 2.4 DNA extraction and molecular identification of sand flies

The preserved parts, the thorax and abdomen, of the remaining 400 female sand flies were separately homogenized in 180 μl of lysis buffer GL using sterile pestles prior to genomic DNA extraction using ISOLATE II Genomic DNA Kit (Bioline, Eveleigh, Australia) according to the manufacturerʼs instructions. The obtained genomic DNA extracts were stored at −20 °C for later use.

PCR amplification targeting the mitochondrial cytochrome *c* oxidase subunit 1 (*cox*1) gene (Table 1) was conducted to verify the morphological identification. PCR amplifications were carried out in 20-μl reaction volumes comprising 4 μl of ready-to-load 5× HOT FIREpol Blend mastermix (Solis BioDye, Tartu, Estonia), 1 μl of 10 pmol of each primer, 2 μl of 2.5–7.0 ng of template DNA, and 12 μl nuclease-free water (Sigma, St. Louis, MO, USA). Amplification PCR conditions comprised of an initial denaturation step at 95 °C for 15 min, followed by 35 cycles of denaturation at 95 °C for 20 s, annealing at 45 °C for 30 s, and extension at 72 °C for 30 s, and a final extension step at 72 °C for 7 min. Amplicons were then resolved in an ethidium bromide-stained 1.5% agarose gel and visualized using Kodak Gel Logic 200 Imaging System (SPW Industrial, Laguna Hills, CA, USA).

**Table 1:** Primers used in this study.

Target gene/region	Primer name	Sequence (5′-3′)	Organism	Amplicon size (with/without primers) (bp)	Reference
*cox*1	LCO1490	GGTCAACAAATCATAAAGTATTGG	Sand flies	704/658	Folmer et al. (1994)
	HCO2198	TAAACTTCAGGGTGACCAAAAAATCA			
ITS1	L5.8S	TGATACCACTTATCGCACTT	Parasites	358–408/270–320	Owino et al. (2021); Echchakery et al. (2017)
	LITSR	CTGGATCATTTTCCGATG			
*cyt b*	CYT-B F	CCCCTCAGAATGATATTTGTCCTCA	Vertebrate hosts	357/307	Boakye et al. (1999)
	CYT-B R	CATCCAACATCTCAGCATGATGAAA			

### 2.5 PCR amplification of the ITS1 region of the 28S rRNA gene of *Leishmania* spp.

The genomic DNA samples extracted from female sand flies were screened for *Leishmania* parasites using the L5.8S and LITSR primer pair (Table 1) that flank 270–320 bp of the internal transcribed spacer region 1 (ITS1) (Schönian et al., 2003). PCR volumes of 20 μl contained 4 μl of 2× Dream*Taq* Green PCR Mastermix (with a high-fidelity Dream*Taq* DNA polymerase, 2× DreamTaq Green buffer, 0.4 mM each dNTP, and 4 mM MgCl_2_), 1 μl of 10 μM of each primer, 2 μl of the DNA template, and 12 μl nuclease-free water. *Leishmania major* (Friedlin strain) DNA was used as a reference positive control and nuclease-free water was used as a negative control. The PCR cycling conditions were programmed as follows: an initial denaturation at 95 °C for 15 min, followed by 35 cycles of denaturation at 95 °C for 30 s, annealing at 58 °C for 30 s, and extension at 72 °C for 1 min, and a final extension step at 72 °C for 7 min. The final PCR amplicons were then characterized by electrophoresis using ethidium bromide-stained 1.5% agarose gels and visualized using Kodak Gel Logic 200 Imaging System (SPW Industrial, Laguna Hills, CA, USA).

### 2.6 PCR amplification of the *cyt b* gene for blood-meal source identification

The blood-meal sources of the engorged female sand flies were determined through PCR amplification of mitochondrial *cytochrome b* (*cyt b*) gene using the CYT-B forward and reverse primers (Boakye et al., 1999) (Table 1). A total reaction volume of 20 μl was used in the PCR reactions with 4 μl 5× HOT FIREPol® Blend Master Mix (with 7.5 mM MgCl_2_) (Solis BioDyne, Estonia), 1 μl of 10 μM of each primer, 1 μl of the DNA template, and 13 μl nuclease-free water. The DNA obtained from the blood of Balb/c mice from the Animal Care Unit at *icipe* and nuclease-free water (Sigma, St. Louis, USA) were used as the reference positive and negative controls, respectively. The PCR amplifications were carried out using thermocycling conditions involving initial denaturation at 95 °C for 15 min and 35 cycles of denaturation at 95 °C for 30 s, annealing at 58 °C for 30 s, and extension at 72 °C for 30 s, with a final extension step at 72 °C for 7 min. All PCR amplifications were performed in a ProFlex™ 3 × 32-well PCR system (ThermoFisher Scientific, USA). The final *cyt b* amplicons were then resolved in an ethidium bromide-stained 1.5% agarose gel and visualized using Kodak Gel Logic 200 Imaging System (SPW Industrial, Laguna Hills, CA, USA).

### 2.7 Purification of the PCR amplicons and sequencing

The resolved DNA bands corresponding to the appropriate amplicon sizes were gel-purified using ISOLATE II Gel Extraction Kit (Bioline, Eveleigh, UK) according to the manufacturerʼs protocol. Purified PCR products (∼10 μl) were shipped to Macrogen (Netherlands) for Sanger sequencing using the respective forward and reverse primers (Table 1).

### 2.8 Data analyses

The information collected pertaining to sand flies trapping sites, trapping numbers, sand fly numbers, species ID, GPS locations, and trapping dates were entered and analyzed in Microsoft Excel (v. 2016) for sand fly diversity and distribution through quantitative counts of each species per sampling area. Descriptive analysis was also adopted in presenting sand fly composition and distributions. Sand fly diversity was analyzed considering species richness and evenness indices in comparison to Shannon index generated using PAST software (v. 2018) (https://softfamous.com/past/) and SpadeR (Species-richness Prediction and Diversity Estimation in R) program (v. 2019) (https://chao.shinyapps.io/SpadeR/).

The nucleotide sequence chromatograms obtained from sequenced amplicons were edited and aligned with reference sequences obtained from the GenBank nr database using the MAFFT plugin in Geneious Prime software v. 2023.1.2. Each consensus sequence was queried for related reference sequences in the GenBank database using the Basic Local Alignment Search Tool (www.ncbi.nlm.nih.gov/BLAST/). A similarity of over 99% in each queried sequence against the subject sequences at the GenBank database was considered to be the most likely organism. Maximum-likelihood phylogenies of the studied sand fly species based on the 597-bp *cox*1 gene sequences and *Leishmania* spp. based on 174-bp ITS1 sequences, aligned with reference sequences retrieved from GenBank, were constructed using PhyML v. 3.0, with automatic model selection based on the Akaike information criterion (Lefort et al., 2017). Tree topologies were estimated over 1000 bootstrap replicates with the nearest neighbour interchange improvements (Guindon et al., 2010). Phylogenetic trees were visualized using FigTree v. 1.4.4 (Rambaut, 2020). Sequences of *Lucilia cuprina* (KJ129390) and *Crithidia brevicula* (KJ474900) were used as outgroups in the *cox*1 and ITS1 phylogenetic trees, respectively.

## 3 Results

### 3.1 Diversity and distribution of morphologically identified phlebotomine sand flies

A total of 1009 sand flies (*n* = 394 males; *n* = 615 females) were trapped in Tirgamo (*n* = 410; 40.6%), Silapani (*n* = 244; 24.2%), Malgis (*n* = 177; 17.5%), Sakardalla (*n* = 138; 13.7%), and Laisamis Centre (*n* = 40; 4.0%) and morphologically identified (Table 2). These consisted of 4 species of *Phlebotomus* and 11 species of *Sergentomyia*. The most common sand fly species collected was *S. clydei* (*n* = 602; 59.7%). Although we sampled 149 *Ph. alexandri*, representing 14.8% of this studyʼs collections, 90% (*n* = 134) of these were collected in Tirgamo. Other species collected included *Sergentomyia squamipleuris* (10.4% of total collection), *Sergentomyia schwetzi* (3.7% of total collection), *Sergentomyia africanus* (3.0% of total collection), *Sergentomyia affinis* (2.5% of total collection), *Ph. orientalis* (1.7% of total collection), *Ph. saevus* (1.3% of total collection), *Sergentomyia yvonnae* (0.8% of total collection), *Sergentomyia bedfordi* (0.8% of total collection), *Sergentomyia dreyfussi* (0.8% of total collection), *Sergentomyia antennatus* (0.2% of total collection), *Ph. martini* (0.1% of total collection), *Sergentomyia yusafi* (0.1% of total collection), and *Sergentomyia harveyi* (0.1% of total collection). A putative new species of *Sergentomyia* (0.2% of total collection) was also identified based on its unique striated spermathecal walls (see below).

**Table 2:** Sand fly species composition and distribution in five sampled areas of Laisamis Ward.

Sampling areas	Sakardalla			Silapani			Tirgamo			Laisamis Centre			Malgis			Total		Abundance
Sand fly species	F	M	T	F	M	T	F	M	T	F	M	T	F	M	T	F	M	*N* (%)
*Ph. alexandri*	0	0	0	1	1	2	107	27	134	10	3	13	0	0	0	118	31	149 (14.8)
*Ph. martini*	0	0	0	0	0	0	0	1	1	0	0	0	0	0	0	0	1	1 (0.1)
*Ph. orientalis*	2	2	4	0	3	3	3	2	5	0	1	1	3	1	4	8	9	17 (1.7)
*Ph. saevus* (*s.l.*)	0	0	0	0	0	0	0	0	0	0	0	0	3	10	13	3	10	13 (1.3)
*S. affinis*	0	0	0	0	0	0	0	0	0	0	0	0	15	10	25	15	10	25 (2.5)
*S. africanus*	1	1	2	1	1	2	15	10	25	0	0	0	0	1	1	17	13	30 (3.0)
*S. antennatus*	0	0	0	0	0	0	2	0	2	0	0	0	0	0	0	2	0	2 (0.2)
*S. bedfordi*	2	2	4	2	0	2	2	0	2	0	0	0	0	0	0	6	2	8 (0.8)
*S. clydei*	36	42	78	124	82	206	88	117	205	9	7	16	72	25	97	329	273	602 (59.7)
*S. dreyfussi*	0	0	0	0	0	0	6	1	7	1	0	1	0	0	0	7	1	8 (0.8)
*S. harveyi*	0	0	0	0	0	0	0	0	0	0	0	0	0	1	1	0	1	1 (0.1)
*S. schwetzi*	10	0	10	1	0	1	3	2	5	5	2	7	7	7	14	26	11	37 (3.7)
*S. squamipleuris*	25	15	40	19	8	27	21	3	24	1	1	2	11	1	12	77	28	105 (10.4)
*S. yusafi*	0	0	0	0	1	1	0	0	0	0	0	0	0	0	0	0	1	1 (0.1)
*S. yvonnae*	0	0	0	0	0	0	0	0	0	0	0	0	6	2	8	6	2	8 (0.8)
*Sergentomyia* sp*.*^a^	0	0	0	0	0	0	0	0	0	0	0	0	1	1	2	1	1	2 (0.2)
Total	76	62	138	148	96	244	247	163	410	26	14	40	118	59	177	615	394	1009 (100)

*Abbreviations*: F, females; M, males; T, total; N, number.

a Putative new species.

Analysis of the sand fly diversity trapped showed moderate species diversity (Shannon-Weiner index = 1.429 and Evenness = 0.515), also in each of the sampling areas (Table 3), and the species compositions of sampling areas shared high similarity indices (Supplementary Table S2). Sand flies of the genus *Sergentomyia* were more common (82.2%, *n* = 829) than sand flies of the genus *Phlebotomus* (17.8%, *n* = 180). However, both genera were found in all areas. Generally, only *Ph. orientalis* was present across all the sampling areas, including Malgis. *Phlebotomus alexandri* was found in highest numbers (*n* = 134) in Tirgamo, as well as in Laisamis Centre (*n* = 13) and Silapani (*n* = 2). Only one male *Ph. martini* was found in Tirgamo and all *Ph. saevus* (*n* = 13) were found in the rocky hills of Malgis.

**Table 3:** Species richness (S), Shannonʼs index, Simpsonʼs index, dominance, Brillouin’s index and evenness values of the sand flies in the sampling areas of Laisamis Ward, Marsabit County.

Diversity indices	Sakardalla	Silapani	Tirgamo	Laisamis Centre	Malgis
No. of taxa (S)	6	8	10	6	10
No. of individuals	138	244	410	40	177
Dominance (D)	0.401	0.730	0.363	0.282	0.336
Simpson (1-D)	0.599	0.270	0.637	0.718	0.664
Shannon (H)	1.141	0.571	1.281	1.309	1.490
Evenness (e^∧^H/S)	0.522	0.221	0.360	0.617	0.444
Brillouin	1.092	0.546	1.251	1.197	1.427

### 3.2 Morphological characteristics of the newly collected sand flies

#### 3.2.1 *Phlebotomus(Artemievus)alexandri* Sinton, 1928

**Fig. 3:**
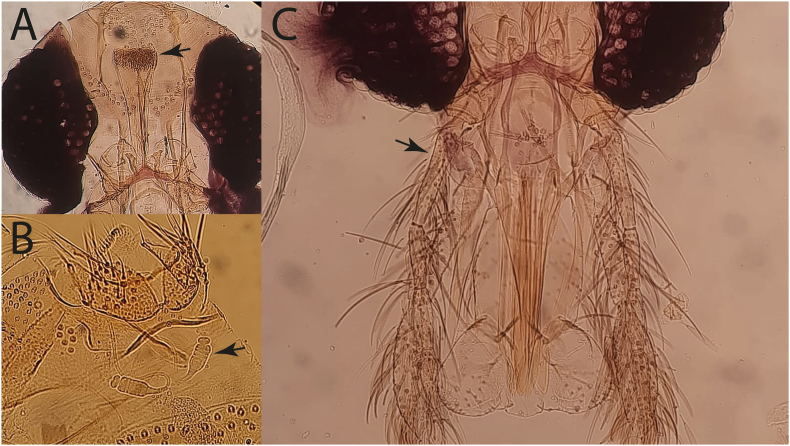
Morphological characteristics of the female *Phlebotomus* (*Artemievus*) *alexandri*. **A** An almost rectangular pharyngeal armature (arrow) with a straight-like line posteriorly. **B** Spermathecae (*arrow*) of segments 8–9 with distinct distal segments. **C** Short antennal segment A3 (*arrow*).

**Fig. 4:**
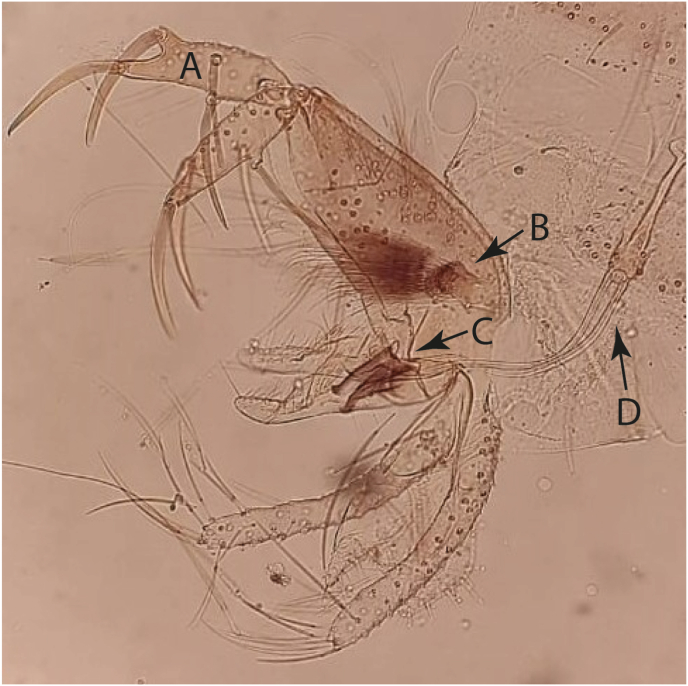
Morphological characteristics of a male *Phlebotomus* (*Artemievus*) *alexandri* terminal genitalia. Gonostyle with four spines (A), short round-headed gonocoxite with numerous projections of setae that are curved at the apex (B), aedeagus curved at the apex (C), and short ejaculatory duct (D).

We morphologically identified females of *Ph. alexandri* Sinton, 1928 based on the unique darkish and thick, stouted pharyngeal armature of almost rectangular shape, nearly straight-lined posteriorly (Fig. 3A), a segmented spermathecae of segments 8–9 with distinctive separate distal segments (Fig. 3B), and a short (half-length of labrum) antennal segment A3 (Fig. 3C), as also found in *Phlebotomus mireillae*. As in members of the subgenus *Paraphlebotomus*, males of *Ph. alexandri* possessed 4 gonostyle spines with apical spines in each gonostyle of almost unequal size, small or short round-headed gonocoxite tuft or sub-basal process with numerous dark-brownish projections of setae that are of unequal length and curved on apex. Moreover, males also had a short ejaculatory duct with a small ejaculatory pump head connected to a short dark-brown adeagus that is curved upwards on the apex as shown in Fig. 4. The paramere was almost twice as long as the adeagus. The pharynx was characteristically ridged as in the members of subgenus *Paraphlebotomus*, *Ph. saevus* and *Ph. mireillae*.

#### 3.2.2 *Sergentomyia yvonnae* Parrot & Schwetz, 1937

**Fig. 5:**
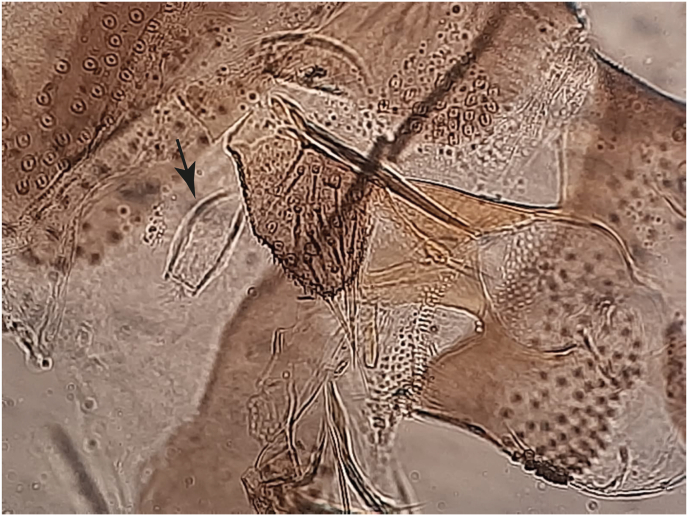
An image of spermathecal capsule (*arrow*) of *Sergentomyia yvonnae* collected in rock crevices at Malgis, Marsabit County, showing unique hair projections that originate from almost the same point of the capsule at the center of the almost straight-lined posterior wall.

We morphologically identified females of *S. yvonnae* based on the major characteristics of this species that include long antennal segment A3, which is longer or equal to the labrum length, and a spermathecal capsule with a unique hair projection that originates on an almost straight-lined part (middle) posteriorly (Fig. 5). The specimens also exhibited 9–10 irregular vertical darkish cibarial teeth on a well-developed darkish pigment plate and the pharynx was characteristically ridged with small few denticles and narrowed posteriorly. The males collected along with females of this species also had long antennal segments A3 with 4 terminally positioned spines, and a long straight dark-brown aedeagus.

#### 3.2.3 *Sergentomyia* sp., a putative new species of sand fly

**Fig. 6:**
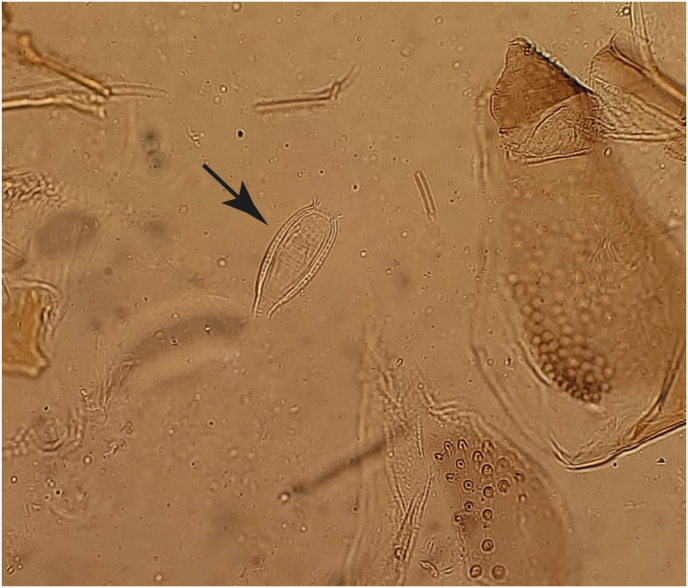
An image of striated bi-walled spermathecal capsule (*arrow*) of the putative new species of *Sergentomyia*.

A putative new sand fly species, *Sergentomyia* sp., was morphologically identified and distinguished from other sand fly species possessing capsule-like spermathecae (*Sergentomyia garnhami*, *S.* *africanus* and *S. yvonnae*) by its unique striated bi-walled spermathecal capsule (Fig. 6). The posterior part of the spermathecae had numerous hairs of almost equal size, each separately originating from an almost curved surface or almost straight lined posteriorly. The pharynx had characteristically narrowed anterior and posterior ends and few denticles pointing towards the anterior while the cibarium was armed with small 5–9 scattered teeth and a small cibarium pigment plate. The antennal segment A3 was longer or equal to the labrum length. The males of this species had 5 spines on the gonostyle (3 merely separated apical and 2 sub-apical), and bi-walled ejaculatory ducts characterized by striations posteriorly. Antennal segment A3 was also longer or equal to labrum length.

### 3.3 Molecular identification of sand flies

To confirm the species identity of the sand flies, we sequenced the *cox*1 barcode gene (GenBank: ON714140-ON7141164) for a set of representative female specimens. In total, sequences were generated for 10 species: *Ph. alexandri*, *Ph. orientalis*, *Ph. saevus*, *S. affinis*, *S. africanus*, *S. dreyfussi*, *S. schwetzi*, *S. squamipleuris*, *S. yvonnae*, and the putative new species of *Sergentomyia*. Since all male sand flies were wholly mounted onto slides for morphological identification, only females were preserved, and *cox*1 sequenced. Therefore, *Ph. martini*, *S. yusafi* and *S. harveyi* were not sequenced and included in the phylogenetic analysis.

**Fig. 7:**
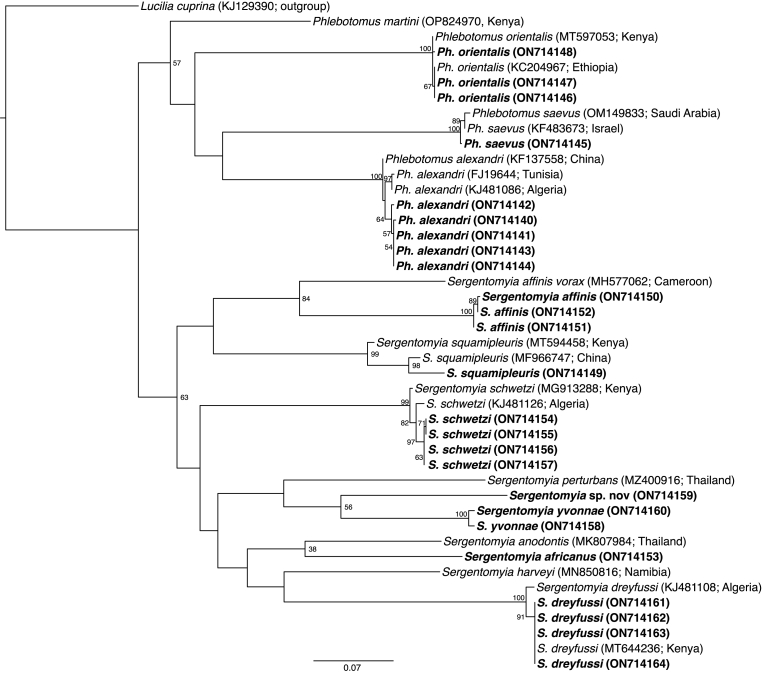
Maximum likelihood phylogeny of sand flies based on *cox*1 gene sequences (597 bp). Representative sequences obtained from this study, with their GenBank accession numbers, are indicated in bold. GenBank accessions and country of origin are indicated for all reference sequences. Bootstrap values at the major nodes are of percentage agreement among 1000 bootstrap replicates. The scale represents the number of substitutions per site. *Lucilia cuprina* (KJ129390) was used as the outgroup.

Phylogenetic analysis of the *cox*1 alignment confirmed morphological identifications of the sand flies, including that of the putative new species of *Sergentomyia* (GenBank: ON714159), which is phylogenetically related to the newly generated sequences for *S. yvonnae* (GenBank: ON714158 and ON714160) (Fig. 7). The sequences of the newly recorded *Ph.* (*Artemievus*) *alexandri* (GenBank: ON714140-ON714144) in this study shared 100% identity with sequences for *Ph. alexandri* from Algeria (GenBank: KJ481088), Tunisia (GenBank: FJ196443) and China (GenBank: KF137558). The *cox*1 sequences of the related *Ph. saevus* (GenBank: ON714145) and *Ph. orientalis* (GenBank: ON714146-ON714148) of the present collection also shared high similarity to corresponding DNA sequences in the GenBank database from both Saudi Arabia (GenBank: OM149833) and Israel (GenBank: KF483673) (*Ph. saevus*) and from Ethiopia (GenBank: KC204967) and Kenya (GenBank: MT597053) (*Ph. orientalis*) (Fig. 7).

Sequences of *S. dreyfussi* were highly similar to corresponding GenBank sequences from Algeria (GenBank: KJ481108) and Kenya (GenBank: MT644236), also distantly phylogenetically related to GenBank sequence of *S. harveyi* (GenBank: MN850816) from Namibia, while those of *S. schwetzi* were closely related to those sequenced from Algeria (GenBank: KJ481126) and Kenya (GenBank: MG913288). Sequences of *S. squamipleuris* showed close relation to those sequenced from China (GenBank: MF966747) and Japan (GenBank: MT694458). Although we found no reference sequence of *S. africanus* in the GenBank database, the newly generated *S. africanus* sequence (GenBank: ON714153) was distantly related to a sequence of *Sergentomyia anodontis* (GenBank: MK807984) from Thailand, while sequences of *S. affinis* were related to GenBank sequence of *S. affinis vorax* (GenBank: MH577062) from Cameroon.

### 3.4 PCR amplification of ITS1 for *Leishmania* spp.

Out of the 400 female sand flies screened for *Leishmania* DNA, 43 sampled from Sakardalla, Silapani and Tirgamo were positive (Table 4). Representative *Leishmania* ITS1 sequences generated during the present study are available under GenBank accessions ON731770-ON731776. Overall occurrence rates of 4.75% (*n* = 19) and 6.00% (*n* = 24) were recorded for *L. major* and *L. adleri*, respectively. The highest occurrence rates of *L. major* DNA were found in *S. clydei* (5.63%; *n* = 12/213) and *Ph. alexandri* (5.19%; *n* = 4/77), while each of *S. africanus*, *S. dreyfussi* and *S. squamipleuris* had single specimens with *L. major* DNA. *Leishmania adleri* DNA occurrence rates were also higher in *S. clydei* (7.51%; *n* = 16/213) and *Ph. alexandri* (2.60%; *n* = 2/7), as well as in *S. squamipleuris* (8.00%; *n* = 4/50). We also found *L. adleri* DNA in single specimens of each of *S. africanus* and *S. schwetzi*.

**Table 4:** *Leishmania* spp. and other trypanosomatid parasites detected in phlebotomine sand flies by PCR amplification of the ITS1 fragment of the 28S rRNA gene (% occurrence).

Sampling areas	Parasites	*Ph. alexandri*	*S. clydei*	*S. africanus*	*S. dreyfussi*	*S. schwetzi*	*S. squamipleuris*	Total
Sakardalla	*L. adleri*	0	6 (18.18)	0	0	0	3 (13.64)	9 (13.04)
Silapani	*L. major*	0	7 (7.37)	0	0	0	1 (10.00)	8 (7.34)
	*L. adleri*	0	10 (10.53)	0	0	1 (100)	1 (10.00)	12 (11.01)
	*Trypanosoma* sp.	0	1 (1.05)	0	0	0	0	1 (0.92)
Tirgamo	*L. major*	4 (5.19)	5 (10.42)	1 (10.00)	1 (25.00)	0	0	11 (7.24)
	*L. adleri*	2 (2.60)	0	1 (10.00)	0	0	0	3 (1.97)
	*Trypanosoma* sp*.*	0	0	1 (10.00)	0	0	1 (8.33)	2 (1.32)

% occurrence per sand fly species		*n* = 77	*n* = 213	*n* = 12	*n* = 5	*n* = 18	*n* = 50	*n* = 400

Parasites	*L. major*	5.19	5.63	8.33	20	–	2.00	4.75
	*L. adleri*	2.60	7.51	8.33	–	5.56	8.00	6.00
	*Trypanosoma* sp.	–	0.47	8.33	–	–	2.00	0.75

Additionally, we detected a 274-bp ITS1 sequence of a *Trypanosoma* sp. (GenBank: ON731777) in a *S. squamipleuris* collected in Tirgamo that shared 100% identity with a *Trypanosoma* sp. (GenBank: MT548851) previously found in *S. squamipleuris* in Kenya. Amplicons of the same length were identified by gel electrophoresis in single samples of *S. clydei* and *S. africanus* from Silapani and Tirgamo, respectively, but were unfortunately not sequenced (Table 4).

**Fig. 8:**
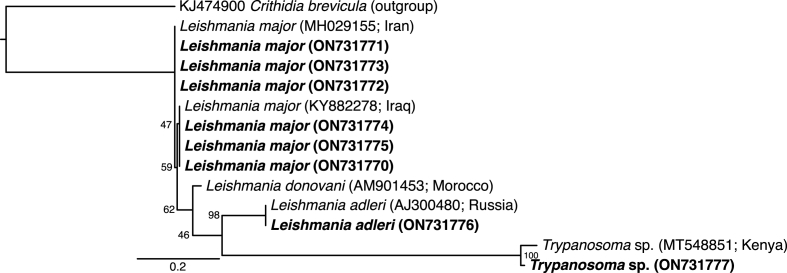
Maximum likelihood phylogeny of *Leishmania* spp. based on ITS1 region of the 28S rRNA gene sequences (174 bp). Representative sequences obtained from this study, with their GenBank accession numbers, are indicated in bold. GenBank accessions and country of origin are indicated for all reference sequences. Bootstrap values at the major nodes are of percentage agreement among 1000 bootstrap replicates. The scale represents the number of substitutions per site. *Crithidia brevicula* (KJ474900) was used as the outgroup.

Phylogenetically, *L. major* sequences (GenBank: ON731770-ON731775) shared 100% nucleotide sequence identity with *L. major* sequenced from Iraq (GenBank: KY882278) and Iran (GenBank: MH029155) (Fig. 8). Also, distinctively different from *L. donovani* (GenBank: AM901453 sequenced from Morocco), one sample of *L. adleri* (GenBank: ON731776) and *Trypanosoma* sp*.* (GenBank: ON731777) each were similar to their corresponding GenBank sequences of *L. adleri* (GenBank: AJ300480) and *Trypanosoma* sp*.* (GenBank: MT548851) from Russia and Kenya, respectively.

### 3.5 Sand fly blood-meal sources

Of the 615 female sand flies trapped, 13 were blood-fed. The *cyt b* sequences of the 9 blood-fed sand flies showed that they had fed on humans (100% nucleotide identity with *Homo sapiens cyt b* (GenBank: MT568794 and MT568795). The blood-meal host sources of the other four blood-fed sand flies could not be determined due to poor amplification. The human *cyt b* sequences obtained from the present blood-meal analysis are available in GenBank under the accession numbers ON720953-ON720961. The human blood-fed sand flies were *Ph. alexandri* (*n* = 4) from Tirgamo, *S. clydei* from Silapani (*n* = 3) and Sakardalla (*n* = 1), and *S. schwetzi* (*n* = 1) from Sakardalla (Table 5)*.* Out of the nine sandflies identified with human blood meals, three had *L. major* DNA (Table 5).

**Table 5:** Distribution of blood-fed sand flies per sampling area (% with *L. major* DNA).

Sampling areas	*Ph. alexandri*	*S. clydei*	*S. schwetzi*	Total
Sakardalla	0	1 (100)	1	2 (50.0)
Silapani	0	3	0	3
Tirgamo	4 (50.0)	0	0	4 (50.0)
Total	4 (50.0)	4 (25.0)	1	9 (33.3)

## 4 Discussion

Our findings show that Laisamis Ward has diverse sand fly species, including four key vectors of leishmaniasis (*Ph. alexandri*, *Ph. martini*, *Ph. orientalis* and *Ph. saevus*). Moreover, we used molecular tools to detect *L. major* DNA in *Ph. alexandri*, *S. africanus*, *S. clydei*, *S. dreyfussi*, and *S. squamipleuris*, thus expanding the number of sand fly species that may potentially contribute to CL transmission in Kenya. Collected sand flies had fed mostly on humans and could be involved in the zoonotic transmission of *Leishmania* spp., including the agents of CL.

Our study also highlights the occurrence of the recently reclassified *Artemievus* sand fly *Ph.* (*Artemievus*) *alexandri* (Cruaud et al., 2021) for the first time in Kenya. *Phlebotomus alexandri* is a vector of VL in some countries of the Middle East (Iran), Mediterranean (Spain) and Asia (northwestern China). Its presence has also been documented in some countries in north and northeastern Africa including Morocco, Djibouti and Ethiopia (Colacicco-Mayhugh et al., 2010). We found *L.* *major* DNA in *Ph. alexandri*, which was surprising, though it had been earlier considered to be an important vector of CL in the former southern U.S.S.R. and a suspected vector of CL in Tunisia and Iran (Lewis, 1982). Therefore, the presence of *Ph. alexandri* in Laisamis (Marsabit County) in northern Kenya raises concerns that this species could be contributing to transmission of VL and CL in the area.

Other sand flies highlighted for their previously unrecognized presence in Kenya include the newly collected putative new species of *Sergentomyia* and *S. yvonnae*. Although the two sand fly species are clearly distinct phylogenetically, based on sequences of the *cox*1 gene (Fig. 7), it is likely that they belong to the same subgenus as they share similar morphological characteristics such as similar capsular spermathecae, long antennal segment A3, and cibarium teeth with pigment plates.

Our molecular screening of sand flies for *Leishmania* parasite DNA found *L. major* and *L. adleri* DNA in *Ph. alexandri*. To our knowledge, no case of human CL has been reported from the region of study apart from reports of molecular detection of *L. major* DNA in *S. squamipleuris* from the neighboring County of Isiolo (Owino et al., 2021). We also detected *L. major* DNA in *S. squamipleuris*, as well as in *S. africanus* and *S. dreyfussi*. Despite reports of several *Sergentomyia* species infected with agents of VL and CL, including *S. squamipleuris* (*L. major*) in Kenya (Owino et al., 2021), *S. ingrami* and *S. babu* in India, and *S. ingrami* and *S. hamoni* (*L. tropica*) in Ghana (Maia and Depaquit, 2016), only *S. schwetzi* has been demonstrated for *Leishmania* transmission competence (Sadlova et al., 2013). Here, we also point out the prevalence of *L. major* DNA in *S. clydei* in Laisamis, Marsabit County. This could suggest that *S. clydei* could be a potential vector as previously postulated (Ayari et al., 2016). We, therefore, suggest that further investigations are needed to establish *Ph. alexandri* and *S. clydei* transmission competence of these agents of CL. The frequent observation of *L. major* in circulation may indicate that closer attention should be paid to patients presenting with skin lesions to determine the presence of CL.

Sand flies have previously been found with DNA of *Trypanosoma* spp. of reptilian origin, but their potential to transmit trypanosomiasis is not yet understood (Kato et al., 2010; Owino et al., 2021). Our study also highlights the detection of *Trypanosoma* sp. in *S. squamipleuris*, *S. clydei* and *S. africanus*, which could have been acquired by feeding on domestic animals. Knowledge of the host preferences of sand flies under natural conditions is essential in understanding how host choice and blood-feeding behavior of various sand flies influence their vectorial capacity in the endemic areas of leishmaniasis (Abbate et al., 2020). Analysis of the vertebrate *cyt b* gene of the blood-fed sand flies revealed that the two dominant sand fly species, *Ph. alexandri* and *S. clydei*, were feeding on humans and probably lizards, even though lizard blood meals were not detected, due to the detection of *L. adleri*, a parasite of lizards and of reptile origin (Previato et al., 1997), also associated with CL (Coughlan et al., 2017). These two sand fly species could be included, among other vectors of leishmaniasis, as the important sand fly hosts that could provide a direct target in controlling the spreading of leishmaniasis within the region.

*Sergentomyia schwetzi* also fed on humans in this study, and although we did not identify any human blood meals in *Ph. orientalis*, a recent study in eastern Kenya found that this species also feeds on humans in eastern Kenya (Owino et al., 2021). This biting behavior seems to have been enabled by the accessibility of human blood meals due outdoor sleeping behavior of most residents of Tirgamo, Silapani, and Laisamis Centre (Fig. 2A and B), despite nearby animal sheds. The positioning of human dwellings (also referred as manyattas) (Fig. 2A and B), which are temporary houses constructed to surround animal sheds, predispose occupants to bites from anthropophilic sand flies that can easily penetrate the structures. Therefore, detection of human blood meals in these sand fly species shows that they could be anthropophilic, warranting further investigation.

*Phlebotomus alexandri* was collected in highest abundance (89.9% of species’ specimens sampled; *n* = 134/149) in Tirgamo, where *Acacia xanthophloea* and *Acacia seyal* are the major vegetation cover (Fig. 2). Previous reports have indicated that *Acacia* spp. provide accessible nutrients to sand flies (Hassaballa et al., 2021), and the presence of these two *Acacia* spp. may have possibly contributed to the localization of this sand fly species. This is supported by the fact that *Ph. alexandri* was not found in Sakardalla, where *Acacia xanthophloea* was absent and *Acacia seyal* was scarcely distributed. More research is needed to unravel these strong *Acacia*-sand fly associations.

## 5 Conclusions

We report the occurrence of *Ph. alexandri* for the first time in Kenya alongside other three vectors of leishmaniasis, *Ph. orientalis*, *Ph. saevus* and *Ph. martini*, in Laisamis, Marsabit County. Our findings also demonstrate through blood-meal analysis that *Ph. alexandri* feed on humans and could possibly also vector VL in the region along with the known vectors of VL, *Ph. orientalis* and *Ph. martini.* The abundance of *Ph. alexandri* and *S. clydei* and the fact that DNA of the causative agent of CL, *L. major*, was also detected in these species suggests that they could potentially be involved in the transmission of CL locally. The presence of *Ph. martini*, albeit a single specimen, in the region was also surprising considering that termite mounds, the main habitat for *Ph. martini*, were absent. We suggest that more research be done to establish the pre-disposing factors to the anthropophilic nature of these sand flies, the alternative ecology of *Ph. martini*, and the role of *Ph. alexandri* in the transmission of *Leishmania* parasites in poorly investigated leishmaniasis hotspots such as those in northern Kenya.

## Funding

This research was funded in whole, or in part, by the 10.13039/100010269Wellcome Trust grant #107742/Z/15/Z and the UK Foreign, Commonwealth & Development Office, with support from the Developing Excellence in Leadership, Training and Science in Africa (DELTAS Africa) programme. SK, JV and DMM were supported by Application of Novel Transgenic Technology & Inherited Symbionts to Vector Control (ANTI-VeC) (Grant Number: AV/PP0018/1), funded by the UK Government Global Challenges Research Fund (GCRF). Additional support was obtained from *icipe* institutional funding from the Swedish International Development Cooperation Agency (SIDA), the Swiss Agency for Development and Cooperation (SDC), the Federal Democratic Republic of Ethiopia, and the 10.13039/100013987Government of the Republic of Kenya. The funders had no role in study design, data collection and analysis, decision to publish, or preparation of the manuscript.

## Ethical approval and consent to participate

We obtained approval for this study from the Kenyatta National Hospital-University of Nairobi Ethics Review Committee (KNH-UON ERC) under protocol number P422/10/2011. This approval covered the following key aspects of the study: (i) collection of information from villagers; (ii) sampling of sand flies; and (iii) community engagement on sand flies and vector-borne transmission of leishmaniasis. Permission to collect sand flies in the sampling areas was sought from homeowners and village elders through verbal consent, as most residents were unable to read or write.

## CRediT authorship contribution statement

**Steve Kiplagat:** Conceptualization, Methodology, Data curation, Validation, Formal analysis, Investigation, Visualization, Writing - original draft, Writing - review & editing. **Jandouwe Villinger:** Methodology, Data curation, Formal analysis, Resources, Visualization, Supervision, Project administration, Funding acquisition, Writing - review & editing. **Collins K. Kigen:** Methodology, Data curation, Formal analysis, Investigation. **Kevin O. Kidambasi:** Methodology, Data curation, Formal analysis, Investigation, Writing - review & editing. **Jackson M. Muema:** Methodology, Data curation, Formal analysis, Investigation, Writing - review & editing. **Stephie M. Mwangi:** Methodology, Investigation. **Maureen Wangari:** Methodology, Investigation**. Damaris Matoke-Muhia:** Writing - review & editing. **Daniel K. Masiga:** Resources, Funding acquisition, Writing - review & editing. **Joel L. Bargul:** Conceptualization, Methodology, Data curation, Formal analysis, Investigation, Resources, Supervision, Project administration, Funding acquisition, Writing - original draft, Writing - review & editing.

## Declaration of competing interests

The authors declare that they have no known competing financial interests or personal relationships that could have appeared to influence the work reported in this paper.

## Data Availability

All data generated or analyzed during this study are included in this published article and its supplementary files. The sequences generated in this study have been deposited in the GenBank database under the accession numbers ON714140-ON7141164 (sand fly *cox*1 gene sequences), ON731770-ON731775 (*Leishmania major* ITS1 sequences), ON731776 (*Leishmania adleri* ITS1 sequence), ON731777 (*Trypanosoma* sp. ITS1 sequence), and ON720953-ON720961 (vertebrate blood meal *cyt b* gene sequences).

## References

[bib1] Abbate J.M., Maia C., Pereira A., Arfuso F., Gaglio G., Rizzo M. (2020). Identification of trypanosomatids and blood feeding preferences of phlebotomine sand fly species common in Sicily, southern Italy. PLoS One.

[bib2] Abonnenc E., Minter D.M. (1965). Bilingual keys for the identification of sand flies of the Ethiopian region. Cah. ORSTOM Entomol. Med..

[bib3] Anjili C.O., Ngumbi P.M., Kaburi J.C., Irungu L.W. (2011). The phlebotomine sand fly fauna (Diptera: Psychodidae) of Kenya. J. Vector Borne Dis..

[bib4] Ayari C., Ben Othman S., Chemkhi J., Tabbabi A., Fisa R., Ben Salah A. (2016). First detection of *Leishmania major* DNA in *Sergentomyia* (*Sintonius*) *clydei* (Sinton, 1928, Psychodidae: Phlebotominae), from an outbreak area of cutaneous leishmaniasis in Tunisia. Infect. Genet. Evol..

[bib5] Bargul J.L., Kidambasi K.O., Getahun M.N., Villinger J., Copeland R.S., Muema J.M. (2021). Transmission of ‘Candidatus *Anaplasma camelii*’ to mice and rabbits by camel-specific keds, *Hippobosca camelina*. PLoS Negl. Trop. Dis..

[bib6] Boakye D.A., Tang J., Truc P., Merriweather A., Unnasch T.R. (1999). Identification of bloodmeals in haematophagous Diptera by cytochrome *b* heteroduplex analysis. Med. Vet. Entomol..

[bib7] Chufe J.C., Oindo B.O., Abuom P. (2019). Determination of impacts of drought on pastoral production system in Marsabit, Kenya. Int. J. Humanit. Soc. Sci..

[bib8] Colacicco-Mayhugh M.G., Masuoka P.M., Grieco J.P. (2010). Ecological niche model of *Phlebotomus alexandri* and *P. papatasi* (Diptera: Psychodidae) in the Middle East. Int. J. Health Geogr..

[bib9] Coughlan S., Mulhair P., Sanders M., Schonian G., Cotton J.A., Downing T. (2017). The genome of *Leishmania adleri* from a mammalian host highlights chromosome fission in *Sauroleishmania*. Sci. Rep..

[bib10] Cruaud A., Lehrter V., Genson G., Rasplus J.Y., Depaquit J. (2021). Evolution, systematics and historical biogeography of sand flies of the subgenus *Paraphlebotomus* (Diptera, Psychodidae, *Phlebotomus*) inferred using restriction-site associated DNA markers. PLoS Negl. Trop. Dis..

[bib11] Dulacha D., Mwatha S., Lomurukai P., Owiny M., Matini W., Irura Z. (2019). Epidemiological characteristics and factors associated with visceral leishmaniasis in Marsabit County, Northern Kenya. J. Interv. Epidemiol. Public Health..

[bib12] Echchakery M., Chicharro C., Boussaa S., Nieto J., Carrillo E., Sheila O. (2017). Molecular detection of *Leishmania infantum* and *Leishmania tropica* in rodent species from endemic cutaneous leishmaniasis areas in Morocco. Parasites Vectors.

[bib13] Elaagip A., Ahmed A., Wilson M.D., Boakye D.A., Abdel Hamid M.M. (2020). Studies of host preferences of wild-caught *Phlebotomus orientalis* and *Ph. papatasi* vectors of leishmaniasis in Sudan. PLoS One.

[bib14] Elnaiem D.-E.A. (2011). Ecology and control of the sand fly vectors of *Leishmania donovani* in East Africa, with special emphasis on *Phlebotomus orientalis*. J. Vector Ecol..

[bib15] Fernández M.S., Santini M.S., Diaz J.I., Villarquide L., Lestani E., Salomón O.D. (2016). Parasitism by tylenchid nematodes in atural populations of *Pintomyia fischeri* (Diptera: Psychodidae: Phlebotominae) in Argentina. SM Trop. Med. J..

[bib16] Folmer O., Black M., Hoeh W., Lutz R., Vrijenhoek R. (1994). DNA primers for amplification of mitochondrial cytochrome *c* oxidase subunit I from diverse metazoan invertebrates. Mol. Mar. Biol. Biotechnol..

[bib17] Guindon S., Dufayard J.F., Lefort V., Anisimova M., Hordijk W., Gascuel O. (2010). New algorithms and methods to estimate maximum-likelihood phylogenies: Assessing the performance of PhyML 3.0. Syst. Biol..

[bib18] Hassaballa I.B., Sole C.L., Cheseto X., Torto B., Tchouassi D.P. (2021). Afrotropical sand fly-host plant relationships in a leishmaniasis endemic area, Kenya. PLoS Negl. Trop. Dis..

[bib19] Inceboz T., Rodriguez-Morales A.J. (2019). Current Topics in Neglected Tropical Diseases.

[bib20] Jones C.M., Welburn S.C. (2021). Leishmaniasis beyond East Africa. Front. Vet. Sci..

[bib21] Kanyina E.W. (2020). Characterization of visceral leishmaniasis outbreak, Marsabit County, Kenya, 2014. BMC Publ. Health.

[bib22] Kato H., Uezato H., Sato H., Bhutto A., Soomro F., Baloch J. (2010). Natural infection of the sand fly *Phlebotomus kazeruni* by *Trypanosoma* species in Pakistan. Parasites Vectors.

[bib23] Khan K., Wahid S., Khan N.H., Ali N. (2017). Potential resting and breeding sites of sand flies (Diptera: Psychodidae) and their habitat characteristics in leishmaniasis foci of Dir districts, Khyber Pakhtunkhwa, Pakistan. J. Med. Entomol..

[bib24] KNBS (2013). https://www.scirp.org/(S(351jmbntvnsjt1aadkposzje))/reference/ReferencesPapers.aspx?ReferenceID=1170085.

[bib25] KSPC (2021). https://www.health.go.ke/wp-content/uploads/2021/07/KSPC-OF-LEISHMANIASIS-STRATEGY-2021-2025.pdf.

[bib26] Lefort L., Longueville J.-E., Olivier Gascuel O. (2017). SMS: Smart Model Selection in PhyML. Mol. Biol. Evol..

[bib27] Lewis D. (1982). A taxonomic review of the genus *Phlebotomus* (Diptera: Psychodidae). Bull. Br. Mus. (Nat. Hist.).

[bib28] Maia C., Depaquit J. (2016). Can *Sergentomyia* (Diptera, Psychodidae) play a role in the transmission of mammal-infecting *Leishmania*?. Parasite.

[bib29] Mann S., Frasca K., Scherrer S., Henao-Martínez A.F., Newman S., Ramanan P. (2021). A review of leishmaniasis: Current knowledge and future directions. Curr. Trop. Med. Rep..

[bib30] Ngere I., Gufu Boru W., Isack A., Muiruri J., Obonyo M., Matendechero S. (2020). Burden and risk factors of cutaneous leishmaniasis in a peri-urban settlement in Kenya. PLoS One.

[bib31] Ngumbi P.M., Kaburi J.C., Anjili C.O., Haas F. (2010). *Phlebotomus* (*Larroussius*) *orientalis* (Diptera: Psychodidae) as a probable secondary vector of visceral leishmaniasis in Kenya. J. Vector Borne Dis..

[bib32] Owino B.O., Matoke-Muhia D., Alraey Y., Mwangi J.M., Ingonga J.M., Ngumbi P.M. (2019). Association of *Phlebotomus guggisbergi* with *Leishmania major* and *Leishmania tropica* in a complex transmission setting for cutaneous leishmaniasis in Gilgil, Nakuru County, Kenya. PLoS Negl. Trop. Dis..

[bib33] Owino B.O., Mwangi J.M., Kiplagat S., Mwangi H.N., Ingonga J.M., Chebet A. (2021). Molecular detection of *Leishmania donovani, Leishmania major*, and *Trypanosoma* species in *Sergentomyia squamipleuris* sand flies from a visceral leishmaniasis focus in Merti sub-County, eastern Kenya. Parasites Vectors.

[bib34] Previato J.O., Jones C., Wait R., Routier F., Saraiva E., Mendonça-Previato L. (1997). *Leishmania adleri* a lizard parasite, expresses structurally similar glycoinositolphospholipids to mammalian *Leishmania*. Glycobiology.

[bib35] Rambaut A. (2020).

[bib36] Sadlova J., Dvorak V., Seblova V., Warburg A., Votypka J., Volf P. (2013). *Sergentomyia schwetzi* is not a competent vector for *Leishmania donovani* and other *Leishmania* species pathogenic to humans. Parasites Vectors.

[bib37] Sang D.K., Ndegwa C.W., Okelo G.B.A., Ashford R.W. (1993). New foci of cutaneous leishmaniasis in Central Kenya and the Rift Valley. Trans. R. Soc. Trop. Med. Hyg..

[bib38] Schönian G., Nasereddin A., Dinse N., Schweynoch C., Schallig H.D.F.H., Presber W. (2003). PCR diagnosis and characterization of *Leishmania* in local and imported clinical samples. Diagn. Microbiol. Infect. Dis..

[bib39] Torres-Guerrero E., Quintanilla-Cedillo M.R., Ruiz-Esmenjaud J., Arenas R. (2017). Leishmaniasis: A review. F1000Research..

[bib40] Tsirigotakis N., Pavlou C., Christodoulou V., Dokianakis E., Kourouniotis C., Alten B. (2018). Phlebotomine sand flies (Diptera: Psychodidae) in the Greek Aegean Islands: Ecological approaches. Parasites Vectors.

[bib41] WHO (2021). https://www.who.int/health-topics/leishmaniasis#tab=tab_1.

[bib42] Zivdari M., Hejazi S.H., Mirhendi S.H., Jafari R., Rastegar H.-A., Abtahi S.M. (2018). Molecular identification of *Leishmania* parasites in sand flies (Diptera, Psychodidae) of an endemic foci in Poldokhtar, Iran. Adv. Biomed. Res..

