# Supplementary material for: Discovery of the vector of visceral leishmaniasis, Phlebotomus (Artemievus) alexandri Sinton, 1928, in Kenya suggests complex transmission dynamics
Source: Curr Res Parasitol Vector Borne Dis. 2023 Jul 28;4:100134. doi: 10.1016/j.crpvbd.2023.100134 (PMC10428034; doi:10.1016/j.crpvbd.2023.100134)
Supplement: Multimedia component 1 [file mmc1.docx]

**Supplementary data**

**Supplementary Table S1.** Coordinates and average altitudes of the study areas in Laisamis sub-county, Marsabit County, Kenya.

| Sub-location | Coordinates | Average Altitude |
| --- | --- | --- |
| Silapani | 1.61478°N, 37.78849°E | 553 m |
| Sakardalla | 1.660026°N, 37.816291°E | 538 m |
| Laisamis center | 1.59642°N, 37.81061°E | 525 m |
| Tirgamo | 1.59769°N, 37.84256°E | 566 m |
| Malgis | 1.835081°N, 37.869628°E | 500 m |

**Supplementary Table S2.** Estimations of pairwise similarities between sampling areas of Laisamis sub-county, Marsabit County, Kenya.

| **Estimated pairwise similarity** | |  |
| --- | --- | --- |
| **Study areas contrast** | **Regional similarity (C12 = U12)** | **Shannon or Horn similarity (q = 1)** |
| 1 *vs* 2 | 0.881 | 0.882 |
| 1 *vs* 3 | 0.930 | 0.930 |
| 1 *vs* 4 | 0.787 | 0.788 |
| 1 *vs* 5 | 0.877 | 0.877 |
| 2 *vs* 3 | 0.887 | 0.889 |
| 2 *vs* 4 | 0.770 | 0.770 |
| 2 *vs* 5 | 0.785 | 0.786 |
| 3 *vs* 4 | 0.793 | 0.793 |
| 3 *vs* 5 | 0.809 | 0.809 |
| 4 *vs* 5 | 0.773 | 0.774 |
| **Average pairwise similarity** | **0.829** | **0.830** |

Study areas: 1, Sakardalla; 2, Silapani; 3, Tirgamo; 4, Laisamis centre; 5, Malgis. C12, Equal-weight Horn measure; U12, Horn size-weighted measure; q, Hill numbers (Shannon diversity order).
